# Cryo-EM structures of SARS-CoV-2 BA.2-derived subvariants spike in complex with ACE2 receptor

**DOI:** 10.1038/s41421-023-00607-2

**Published:** 2023-11-02

**Authors:** Yaning Li, Chang Ren, Yaping Shen, Yuanyuan Zhang, Jin Chen, Jiangnan Zheng, Ruijun Tian, Liwei Cao, Renhong Yan

**Affiliations:** 1 Center for Infectious Disease Research, Westlake Laboratory of Life Sciences and Biomedicine, Key Laboratory of Structural Biology of Zhejiang Province, School of Life Sciences, Westlake University, Hangzhou, Zhejiang China; 2 Tsinghua-Peking Joint Center for Life Sciences, School of Life Sciences, Tsinghua University, Beijing, China; 3 Department of Biochemistry, Key University Laboratory of Metabolism and Health of Guangdong, School of Medicine, Institute for Biological Electron Microscopy, Southern University of Science and Technology, Shenzhen, Guangdong China; 4 Department of Chemistry and Research Center for Chemical Biology and Omics Analysis, School of Science, Southern University of Science and Technology, Shenzhen, Guangdong China; 5 Clinical Center for Molecular Diagnosis and Therapy, the Second Affiliated Hospital of Fujian Medical University, Quanzhou, Fujian China

**Keywords:** Electron microscopy, Molecular biology

Dear Editor,

The ongoing emergence of SARS-CoV-2 Omicron variants continues to pose a significant health threat due to their enhanced transmissibility and immune evasion capabilities^1,2^. Among these Omicron variants, the BA.2 variant and its derived subvariants, such as XBB.1 and BA.2.75, have gained prominence for their high infectivity and immune evasion potential^3^. BA.2 variant diversified into multiple sub-lineages. One branch gave rise to the BA.4 and BA.5 sub-lineages, which share the same Spike (S) protein^4^. The BA.5 sub-lineage further evolved into BF.7 and even BQ.1.1^5^. On another branch, BA.2 also evolved into BA.2.75 and BA.2.10.1, and subsequently, these two subvariants recombined to form the XBB variant, which has now become one of the most prevalent substrains globally (Fig. 1a)^5^.

**Fig. 1:**
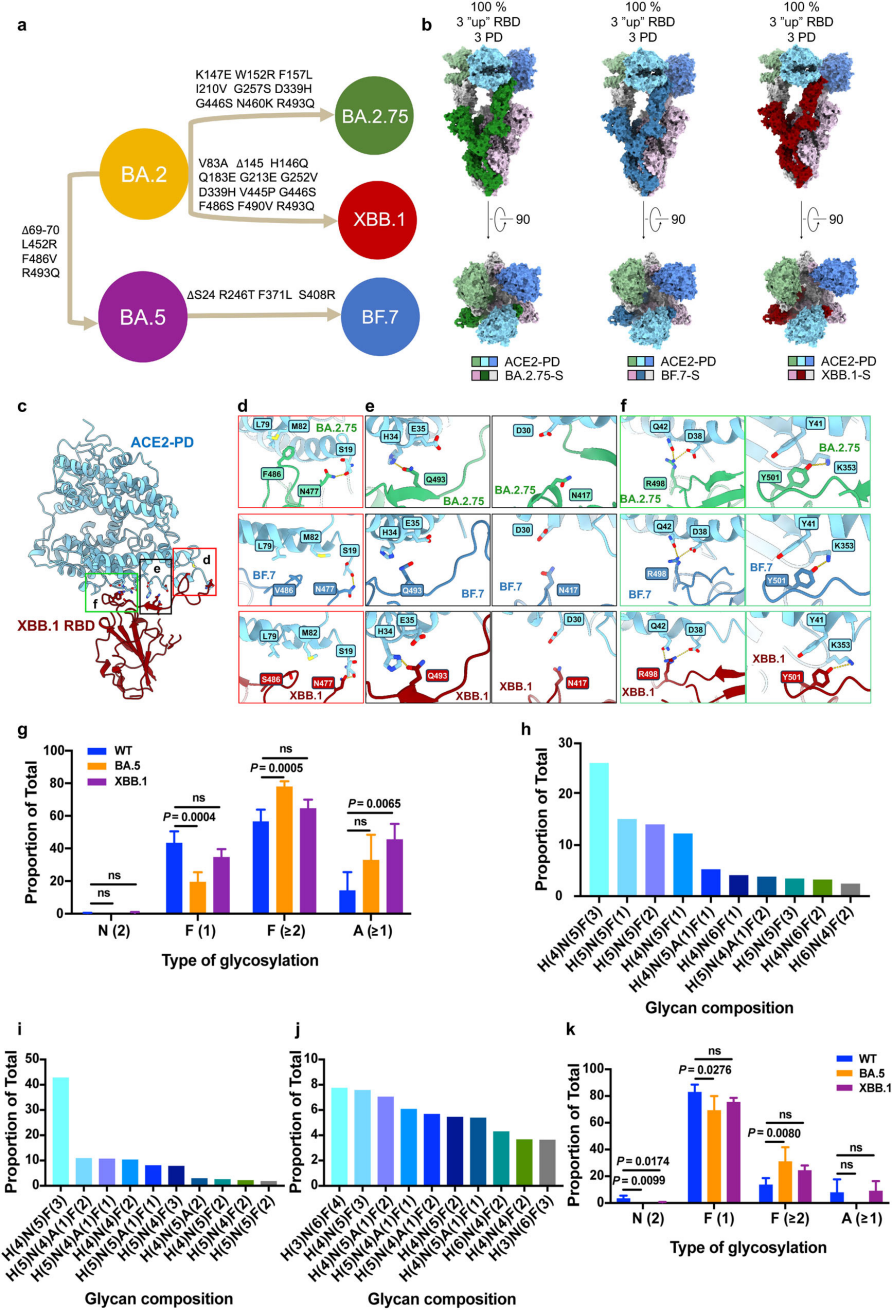
Structural and biological analysis of Omicron subvariants, BA.2.75, BF.7, and XBB.1. **a** Phylogenetic relationships of SARS-CoV-2 subvariants. **b** Surface presentation of domain-colored cryo-EM structures of extracellular domain of S protein (S-ECD) from Omicron BA.2.75, BF.7, and XBB.1 respectively in complex with the PD of ACE2. **c** Representative point mutations of XBB.1 RBD interaction with ACE2. Detailed comparison of the ACE2-interacting residues between BA.2.75, BF.7, XBB.1, and WT RBD are shown in **d**–**f**. **d** S477N of BA.2.75, BF.7, and XBB.1 RBD results in an additional polar interaction with Ser19 of ACE2. F486V of BF.7 remains the hydrophobic interaction with Leu79 and Met82 of ACE2, while F486S in XBB.1 might reduce the hydrophobic interaction. **e** The Q493R in BA.2.75 and XBB.1 leads to a new polar interaction with His34, although K417N weakens the original interaction with ACE2-Asp30. **f** Q498R of BA.2.75, BF.7, and XBB.1 RBD results in an additional polar interaction with Asp38 of ACE2. This may compensate for the lost interactions of Asn501 in the WT S protein with Tyr41 of ACE2. **g** Comparison of N-linked glycosylation profiles derived from RBDs of WT, BA.5, and XBB.1 subvariants. **h**–**j** Top ten glycan compositions that were identified on RBD of WT (**h**), RBD of BA.5 (**i**), RBD of XBB.1 (**j**). **k** Comparison of N-linked glycosylation profiles derived from S proteins of WT, BA.5, and XBB.1 subvariants. H: hexose, N: N-acetylglucosamine, F: fucose, A: sialic acid.

The Omicron sub-lineages harbor multiple mutations in the S protein compared to the original strain (referred to as the WT strain), which mediates receptor recognition and facilitates membrane fusion with the host cells (Supplementary Fig. S1a)^6^. Despite the extensive mutations, all these Omicron subvariants still utilize ACE2 as the host receptor^7^. Binding of the receptor-binding domain (RBD) to the peptidase domain (PD) of ACE2 triggers conformational changes in the trimeric S protein, leading to exposure of the fusion peptide and facilitating membrane fusion with host cells^8^.

The Omicron sub-lineages demonstrate an increased capacity for immune escape. Notably, the Asn343 glycan in the RBD region is recognized by the monoclonal antibody S309. S309 fails to neutralize the BA.2 and BA.4/5 subvariants, but can recover potent neutralization against the BA.2.75 and XBB.1 subvariants^9^. The evolution of glycan moieties on the S protein remains an elusive aspect requiring further investigation.

To elucidate the mechanisms underlying the altered properties of different Omicron subvariants, it is crucial to identify the specific mutations on the S protein responsible for these changes. Hence, we characterized the ACE2-bound S protein structures derived from BF.7, BA.2.75, and XBB.1 subvariants.

The monomeric human ACE2-PD exhibited binding to the RBDs of BA.2, BA.5, BA.2.75, BF.7, and XBB.1 with binding affinity (*K*_D_) of 4.80 ± 0.01, 3.2 ± 0.03, 1.2 ± 0.01, 4.1 ± 0.02, and 16.2 ± 0.03 nM, respectively. These values displayed heterogeneity compared to the WT RBD (12.5 ± 0.04 nM) (Supplementary Fig. S1). The binding affinity for WT and BA.2 subvariants was found to be consistent with previous studies^2,8,10^. While the increased affinity of Omicron BA.2.75 and BF.7 subvariants for ACE2 may partially explain their enhanced transmissibility, it does not fully account for the prevalence of the XBB.1 subvariant.

We then employed single-particle cryo-electron microscopy (cryo-EM) to determine the structures of trimeric S proteins in complex with ACE2-PD. The structures of ACE2-PD in complex with S proteins from BA.2.75, BF.7, and XBB.1 were resolved at overall resolutions of 3.5, 3.9, and 3.9 Å, respectively (Supplementary Figs. S2–S8 and Supplementary Table S1). For simplicity, these complexes will be referred to as BA.2.75-SA, BF.7-SA, and XBB.1-SA, respectively. In these structures, the RBDs of the S protein are all in the “up” conformation and each one binds to one ACE2 molecule (Fig. 1b). To further analyze the RBD-PD interface in detail, we also determined the structures of monomeric RBDs (BA.2.75, BF.7, and XBB.1) bound to the ACE2-SIT1 complex. These structures provided improved resolution of the RBD-PD interface, avoiding the flexibility of RBD, and were resolved at local resolutions of 2.9, 3.2, and 3.6 Å, respectively (Supplementary Figs. S5–S9 and Supplementary Table S1).

In comparison to the BA.2 subvariant, BA.2.75 exhibits additional mutations in the RBD, including D339H, G446S, N460K, and R493Q. Similarly, BF.7 carries mutations F371L, S408R, L452R, F486V, and R493Q compared to BA.2. Besides, XBB.1 displays mutations D339H, V445P, G446S, F486S, F490V, and R493Q when compared to BA.2 (Fig. 1a). Despite the presence of these numerous mutations in the subvariants, our structural analysis reveals a strikingly convergent binding pattern between the RBDs and ACE2.

Of particular note is the single difference among BA.2.75, BF.7, and XBB.1: the substitution of Phe486 in BA.2.75 with Val in BF.7 and Ser in XBB.1. The hydrophilic side chain of Ser in XBB.1 may disrupt the original hydrophobic interactions involving Phe486 of the RBD with Leu79 and Met82 of ACE2, thereby providing an explanation for the compromised binding affinity observed in XBB.1 and its RBD. Additionally, the salt bridge formed by Arg493 of BA.2 with Glu35 of ACE2 is replaced by Gln493 in BA.2.75, BF.7, and XBB.1, reminiscent of the wild-type configuration (Fig. 1c–f). Overall, these mutations remodel the interaction network into a highly convergent mode, despite the presence of diverse mutations within the different subvariants.

The immune evasion properties of Omicron subvariants have been extensively studied, but the glycosylation profiles of these subvariants have received less attention. The glycan shield on the surface of the S protein plays a crucial role in the formation of epitopes targeted by broadly neutralizing antibodies. Specifically, the glycosylation site Asn343 in the RBD region is part of the epitope recognized by the S309 antibody. To investigate the potential impact of Asn343 glycan on antibody neutralization, we characterized the N-linked glycosylation profiles of RBDs and trimeric S proteins from SARS-CoV-2 WT, BA.5, and XBB.1 subvariants. The vast majority of N-linked glycans at Asn343 are complex-type glycosylation with modifications of fucose and/or sialic acid in both RBDs and S proteins across three subvariants (Fig. 1g–j), in line with previous study^11^. Interestingly, significantly higher levels of multi-fucosylation and sialylation were observed in the RBDs of BA.5 and/or XBB.1 compared to the WT. Conversely, mono-fucosylation was significantly decreased in the RBD of BA.5 relative to the WT (Fig. 1g–j). A similar trend was observed in the context of the trimeric S proteins (Fig. 1k and Supplementary Fig. S10). These findings suggest that mutations in the RBD region likely influence the composition of N-linked glycans at Asn343 by altering the accessibility of the glycan processing machinery, which is influenced by the local environment created by these mutations.

During the clinical trial of the Pfizer/BioNTech vaccine, two mRNA candidates encoding the RBD and the trimeric S protein were tested to induce antigen-specific immune responses. Interestingly, a broader range of T-cell responses was observed in the group receiving the S protein compared to the group receiving the RBD alone. To investigate the potential role of glycans in this phenomenon, we compared the N-linked glycan profiles of RBDs to the corresponding S proteins across three subvariants: WT, BA.5, and XBB.1 (Supplementary Fig. S11). Strikingly, we found that levels of multi-fucosylation and/or sialylation at Asn343 were significantly increased in the RBDs relative to the corresponding S proteins across all three subvariants. This suggests that the quaternary structure of the SARS-CoV-2 S protein has an impact on its glycosylation. Such changes in glycosylation have implications for the binding of antibodies that target proteoglycan epitopes. Therefore, when considering RBD mRNA/proteins as vaccine candidates, it is crucial to take into account these variations in glycosylation, as they can impact the interaction between the vaccine-induced antibodies and the S protein.

SARS-CoV-2 variants have emerged as a result of selective pressure from the immune system. Omicron sub-lineages, in particular, harbor a large number of mutations in regions that are targeted by therapeutic antibodies and vaccines, providing a mechanistic basis for their immune evasion. For example, compared to BA.2, BA.2.75, XBB.1, and BF.7 carry additional mutations in the N-terminal domain, suggesting the increasing ability to evade antibodies targeting the N-terminal domain. This speculation has been confirmed through pseudo-typed virus neutralization^5,9^ and flexddG-based interfacial analysis^12^. However, despite these mutations, we have identified a convergent binding pattern between RBD and ACE2 in the BA.2.75, BF.7, and XBB.1 subvariants, suggesting ACE2 mimic monoclonal antibodies might have the most broad-spectrum neutralizing potency. Additionally, our mass spectrometry analysis has revealed that the site Asn343 is occupied by larger glycans with multi-fucosylation and sialylation in BA.5 and XBB.1 subvariants compared to the WT, imposing additional steric constraints for antibody binding to this region. Furthermore, we observed higher levels of glycans with multi-fucosylation and sialylation at Asn343 in RBDs relative to the corresponding S proteins. This suggests that the glycans at this site are more accessible to glycan processing enzymes in the RBDs compared to the S proteins. Understanding and incorporating these glycosylation variations will be important for the development of effective vaccines against SARS-CoV-2.

### Supplementary information

Supplementary Information

## Data Availability

Atomic coordinates and cryo-EM density maps of Omicron BA.2.75 S protein in complex with PD of ACE2 (PDB: 8I9B, whole map: EMD-35264), the interface between BA.2.75 RBD and ACE2 (PDB: 8I9F, whole map: EMD-35269), BF.7 S protein in complex with PD of ACE2 (PDB: 8I9C, whole map: EMD-35266), the interface between BF.7 RBD and ACE2 (PDB: 8I9G, whole map: EMD-35270), XBB.1 S protein in complex with PD of ACE2 (PDB: 8I9D, whole map: EMD-35267) and the interface between XBB.1 RBD and ACE2 (PDB: 8I9H, whole map: EMD-35272) have been deposited to the Protein Data Bank (http://www.rcsb.org) and the Electron Microscopy Data Bank (https://www.ebi.ac.uk/pdbe/emdb/), respectively.
