# Supplementary material for: Cryo-EM structures of SARS-CoV-2 BA.2-derived subvariants spike in complex with ACE2 receptor
Source: Cell Discov. 2023 Nov 2;9:108. doi: 10.1038/s41421-023-00607-2 (PMC10622580; doi:10.1038/s41421-023-00607-2)
Supplement: Supplementary file 1 — Supplementary Information [file 41421_2023_607_MOESM1_ESM.pdf]

Materials and methods

Protein expression and purification

The extracellular domain (ECD) (1-1208 a.a) of Spike protein (S protein) of SARS-CoV-2 Omicron variant BA.2.75, BF.7 and XBB.1 were cloned into the pCAG vector (Invitrogen) with six proline substitutions at residues 817, 892, 899, 942, 986 and 987 and a C-terminal T4 fibritin trimerization motif followed by 10×His tag, respectively. A "GSAS" mutation at residues 682 to 685 was introduced into ECD to prevent the host furin protease digestion. These constructs were hereafter referred to as BA. 2.75-S, BF.7-S and XBB.1-S.

The receptor binding domain (RBD) (319-541 a.a) of S protein from SARS-CoV-2 WT strain and Omicron variant BA.2/5/2.75, BF.7 and XBB.1 were cloned into the pCAG vector (Invitrogen) with an N-terminal signal peptide of secreted luciferase and a C-terminal 6×His tag, respectively. These residue numbers mentioned above are that relative to the spike (WT). The peptidase domain (PD) (19-615 a.a) of human ACE2 was also cloned into the pCAG vector (Invitrogen) with an N-terminal signal peptide of secreted luciferase and a C-terminal Flag tag. The cDNAs for full-length human SIT1 (accession number: NM_020208.3) and ACE2 (accession number: NM_001371415) were subcloned into pCAG respectively. An N-terminal FLAG tag was fused to SIT1, and 10xHis was fused at the C-terminal of ACE2 using a standard two-step PCR. The mutants were generated with a standard two-step PCR-based strategy. All the plasmids used to transfect cells were prepared by GoldHi EndoFree Plasmid Maxi Kit (CWBIO). The recombinant protein was overexpressed using the HEK293F mammalian cells at 37℃ under 5% CO_2 in a Multitron-Pro shaker (Infors, 130 rpm). When the cell density reached 2.0 $\times 10^6$ cells/mL, the plasmid was transiently transfected into the cells. To transfect one liter of cell culture, about 1.5 mg of the plasmid was premixed with 3 mg of polyethylenimines (PEIs) (Polysciences) in 50 mL of fresh medium for 15 mins before adding to cell culture. Cells or medium was collected by centrifugation at 4000×g for 15 mins after sixty hours transfection.

The secreted ECD and RBD of S protein were purified by Ni-NTA affinity resin (Qiagen). The nickel resin loaded was rinsed with the wash buffer 1 containing 25 mM

HEPES (pH 7.0), 500 mM NaCl and washed with wash buffer 2 containing 25 mM HEPES (pH 7.0), 150 mM NaCl and 30 mM imidazole. Protein was eluted by wash buffer 2 plus 270 mM imidazole. Then the Ni-NTA eluent of ECD was subjected to size-exclusion chromatography (Superose 6 Increase 10/300 GL, GE Healthcare) in buffer containing 25 mM HEPES (pH 7.0), 150 mM NaCl. The peak fractions were collected and stored at -80℃. The Ni-NTA eluent of RBD was subjected to size-exclusion chromatography (Superose 6 Increase 10/300 GL, GE Healthcare) in PBS buffer (pH 7.4, gibco) with 0.04% Tween-20. The factions were collected for structural analysis and measurement of RBD binding to human ACE2-PD by biolayer interferometry.

The secreted PD was purified by anti-FLAG M2 affinity resin (Sigma Aldrich). After loading two times, the anti-FLAG M2 resin was washed with the wash buffer 3 containing 25 mM HEPES (pH 7.0), 150 mM NaCl. The protein was eluted with the wash buffer 3 plus 0.2 mg/mL flag peptide. The eluent of PD was then concentrated and subjected to size-exclusion chromatography (Superdex 200 Increase 10/300 GL, GE Healthcare) in buffer containing 25 mM HEPES (pH 7.0), 150 mM NaCl.

The BA.2.75-S, BF.7-S and XBB.1-S was incubated with PD at a molar ratio of about 1:6 for one hour. To remove excessive PD, the mixture was subjected to size-exclusion chromatography (Superose 6 Increase 10/300 GL, GE Healthcare) in buffer containing 25 mM HEPES (pH 7.0), 150 mM NaCl. The peak fractions containing protein complex were collected for EM analysis.

To co-express SIT1 and ACE2, about 0.75 mg plasmids for SIT1 and 0.75 mg plasmids for ACE2 were premixed with 3 mg PEIs in 50 ml of fresh medium for 15 mins before adding to cell culture. The transfected cells were cultured for 48 hours before harvesting. For purification of the SIT1 and ACE2 complex, the cells were collected in buffer containing 25 mM HEPES, pH 7.0, 150 mM NaCl, and three protease inhibitors , aprotinin (1.3 μg/ml, AMRESCO), pepstatin (0.7 μg/ml, AMRESCO), and leupeptin (5 μg/ml, AMRESCO). The membrane fraction was solubilized at 4 °C for 2 hours with 1% (w/v) glyco diosgenin (GDN, Anatrace) and the cell debris was removed by centrifugation at 18,700 g for 45 mins. The supernatant was loaded to anti-FLAG M2

affinity resin (Sigma). After rinsing with the wash buffer 3 containing 25 mM HEPES, pH 7.0, 150 mM NaCl, and 0.01% GDN (w/v), the protein was eluted with wash buffer plus 0.2 mg/ml FLAG peptide. The eluent was further purified by Ni-NTA affinity resin (Qiagen). After eluted with the wash buffer 3 supplemented with 300 mM imidazole, the eluent was then concentrated and incubated with BA.2.75, BF.7 and XBB.1 RBD at a molar ratio of about 1:2.4 for 30 mins. Then the protein mixture was subjected to size-exclusion chromatography (Superose 6 Increase 10/300 GL, GE Healthcare) in buffer containing 25 mM HEPES, pH 7.0, 150 mM NaCl and 0.01% GDN. The peak fractions were collected and concentrated for EM analysis.

Measurement of Omicron subvariants and WT strain RBD binding to human ACE2-PD by biolayer interferometry

The binding between PD and RBD of Omicron subvariants were performed using Octet Red96e (ForteBio), and the RBD of WT strain was measured as well at the same time. The PD of ACE2 was biotinylated using biotinylation kit (Genemore, 1828M) and loaded to octet SA biosensor (Sartorius). The association and dissociation of PD-coated biosensors with different concentrations of RBD of SARS-CoV-2 S protein were recorded in binding buffer (PBS pH 7.4, 0.04% Tween-20). Data was analyzed by Octet Data Analysis HT 12.0 software. Reference sample and reference sensor were subtracted, and K D values were analyzed using a 1:1 global fit model. Data were plotted using Prism V8.0 software (GraphPad).

Cryo-EM sample preparation and data acquisition

The ECD-PD complex was concentrated to ~1.5 mg/mL and SIT1-ACE2-RBD complex was concentrated to ~12 mg/mL and applied to the grids. Aliquots (3.3 μL) of the protein were placed on glow-discharged holey carbon grids (Quantifoil Au R1.2/1.3). The grids were blotted for 3.0 s or 3.5 s and flash-frozen in liquid ethane cooled by liquid nitrogen with Vitrobot (Mark IV, Thermo Fisher Scientific). The prepared grids were transferred to a Titan Krios operating at 300 kV equipped with Gatan K3 detector and GIF Quantum energy filter. Movie stacks were automatically

collected using AutoEMation[1], with a slit width of 20 eV on the energy filter and a defocus range from -1.4 μm to -1.8 μm in super-resolution mode at a nominal magnification of 81,000×. Each stack was exposed for 2.56 s with an exposure time of 0.08 s per frame, resulting in a total of 32 frames per stack. The total dose rate was approximately 50 $e^-/Å^2$ for each stack. The stacks were motion corrected with MotionCor2[ref.2] and binned 2-fold, resulting in a pixel size of 1.087 or 1.095 Å/pixel. Meanwhile, dose weighting was performed[3]. The defocus values were estimated with Gctf[4].

Data processing

The Cryo-EM structure of S protein from BA.1-SA has been solved firstly[5] and identical protocol was applied to the complex of other ECD-PD complex. Particles for S-ECD bound with PD of ACE2 were automatically picked using Relion 3.0.6[ref.6-9] from manually selected micrographs. After 2D classification with Relion, good particles were selected and subject to multiple cycle of heterogeneous refinement without symmetry using cryoSPARC[10]. The good particles were selected and subjected to Local CTF Refinement with C1 symmetry, Non-uniform Refinement, resulting in the 3D reconstruction for the whole structures.

Particles of SIT1-ACE2-RBD complex were also automatically picked using Relion 3.0.6[ref.11-14] from manually selected micrographs. After 2D classification, good particles were selected and subject to cycles of heterogeneous refinement with C1 symmetry. The good particles were selected and subject to homogeneous refinement with C2 symmetry. To further improve the map quality of ACE2-RBD interface, the particles were C2-symmetry expanded and re-extracted at the location of the interface between ACE2 and RBD. The re-extracted dataset was subject to several cycles of 3D classified and focused refinement, resulting in a 3D reconstruction with better quality for ACE2-RBD interface.

The resolution was estimated with the gold-standard Fourier shell correlation 0.143 criterion[15] with high-resolution noise substitution[16]. Refer to Supplementary Figs. S3-S9 and Supplementary Table S1 for details of data collection and processing.

Model building and structure refinement

For model building of the complex of ECD-PD complex, the atomic model of the BA.5 S protein in complex with PD of ACE2 (PDB ID: 7Y21) were used as templates, which were MDFF (molecular dynamics flexible fitting)[17] into the whole cryo-EM map of the complex and the focused-refined cryo-EM map of the RBD-PD sub-complex, respectively.

For model building of the complex of RBD-PD complex, the atomic model of the SIT1-ACE2-BA.5 RBD (PDB ID: 7Y76) was sequence-substituted to the BA. 2.75, BF.7 and XBB.1 RBD in chainsaw and fitted into focused refined maps of ACE2-RBD interface using MDFF.

Each residue was manually checked with the chemical properties taken into consideration during model building. Several segments, whose corresponding densities were invisible, were not modeled. Structural refinement was performed in Phenix[18] with secondary structure and geometry restraints to prevent overfitting. To monitor the potential overfitting, the model was refined against one of the two independent half maps from the gold-standard 3D refinement approach. Then, the refined model was tested against the other map. Statistics associated with data collection, 3D reconstruction and model building were summarized in Table S1.

Proteolytic digestion of monomeric RBDs and trimeric S proteins

Approximately 50 μg of the proteins were denatured with 8 M urea in 25 mM HEPES, 500 mM NaCl (pH 7.0). The denatured proteins were then reduced with 5 mM of dithiothreitol (Sigma) at 200 rpm at 37 ℃ for 1 h and alkylated with 10 mM iodoacetamide (Sigma) at room temperature in the dark for 45 min. The urea concentration was diluted 1:4 with 25 mM HEPES, 500 mM NaCl (pH 7.0). The resulting proteins were divided into two equal aliquots for the following proteolytic digestion.

Trypsin and chymotrypsin. The first aliquot was digested with chymotrypsin (Promega) at an enzyme/substrate ratio of 1:10 (w/w) at 200 rpm at 30 ℃ for 10 h, followed by

trypsin (Sigma) treatment at an enzyme/substrate ratio of 1:10 (w/w) at 200 rpm at 37 ℃ for 16 h. The proteolytic reaction was quenched with 50% formic acid (Sigma) via adjusting pH to <3. The resulting buffer was centrifuged at 16500 rpm for 10 min to remove undigested pellets. The peptides were desalted using Sep-Pak C18 reverse-phase cartridge (Waters).

Chymotrypsin. The second aliquot was digested with chymotrypsin at an enzyme/substrate ratio of 1:10 (w/w) at 200 rpm at 30 ℃ for 10 h. The proteolytic reaction was quenched with 50% formic acid (Sigma) via adjusting pH to <3. The resulting buffer was centrifuged at 16500 rpm for 10 min to remove undigested pellets. The peptides were desalted using Sep-Pak C18 reverse-phase cartridge (Waters).

C18 desalting

The digested peptides were desalted with Sep-Pak C18 reverse-phase cartridge (100 mg, Waters). The cartridge was conditioned with 2×1 mL of acetonitrile (ACN), then 2×1 mL of 50% ACN/0.1% FA, followed by 4×1 mL of 0.1% trifluoroacetic acid (TFA, Sigma). The samples were loaded twice. The column was washed with 3×1 mL of 0.1% TFA, followed by 1 mL of 1% FA. The peptides were eluted with 600 μL of 50% ACN/0.1% FA. The peptides were dried using Speed-Vac (JM technology co.).

Glycopeptide enrichment

The glycopeptides were enriched with Oasis Max extraction cartridge (30 mg, Waters). The cartridge was conditioned with 3×1 mL of ACN, then with 3×1 mL of 100 mM triethylammonium acetate buffer; followed by 3×1 mL of water, and finally with 3×1 mL of 95% ACN/1% TFA. The peptides were loaded twice. The cartridge was washed with 3×1 mL of 95% ACN/1% TFA to remove non-glycosylated peptides. The glycopeptide fraction was eluted with 600 μL of 50% ACN/0.1% FA. The glycopeptides were dried using Speed-Vac (JM technology co.).

Nano LC-MS/MS analysis

The glycopeptides were analyzed on a Fusion Orbitrap Tribrid mass spectrometer (Thermo Fisher Scientific). Approximately 2 μg of the glycopeptides were loaded onto a 20 cm, 100 μm column packed with 1.9 μm C18 resin (Dr. Maisch GmbH), and separated at a flow rate of 250 nL/min on an EASY-nLC 1000 system (Thermo Fisher Scientific). Buffer A and B were 0.1% FA in water and 0.1% FA in ACN. The following gradient was deployed: 0-2 min 3-6% B, 2-62 min 6-30% B, 62-71 min 30-60% B, 71-72 min 60-90% B, 72-77 min 90% B, 77-78 min 90-50% B, and 78-87 min 50% B. The glycopeptides were separated on the column and directly sprayed into the mass spectrometer. The mass spectrometer was operated in a data-dependent mode. The parameters were set as follows: MS1 resolution 60K, scan range 500-2000, AGC target 5E5, maximum injection time 50 ms, dynamic exclusion 45 s, charge inclusion 2-6, cycle time 3 s; MS2 resolution 50K, AGC target 1E5, collision energy 34.

MS data processing

Site-specific identification of N-linked glycopeptides was carried out using pGlyco 3.0 (PMID: 28874712). The parameters were set as follows: fragmentation HCD, human protein database downloaded from Uniprot (in January 2019) including the sequences of monomeric RBDs and trimeric S proteins analyzed in this study, enzyme chymotrypsin (or a combination of trypsin and chymotrypsin), maximal missed cleavage 2, peptide length 6-40, peptide mass 600-4000, fixed modification carbamidomethylation (C +57.022 Da), variable modifications oxidation methionine (M +15.995 Da) and acetylation on protein N-terminus (+42.011 Da), glycan database default human N-linked glycan database, precursor tolerance 10 ppm, fragment tolerance 20 ppm, glycopeptide FDR 0.01. The intensities of glycopeptides were extracted using pGlycoQuant (PMID: 36477196). The parameters were set as follows: type of identification results pGlyco, threshold FDR 0.01, type of quantification DDA label free. The statistical analyses were performed with GraphPad Prism 8.0.

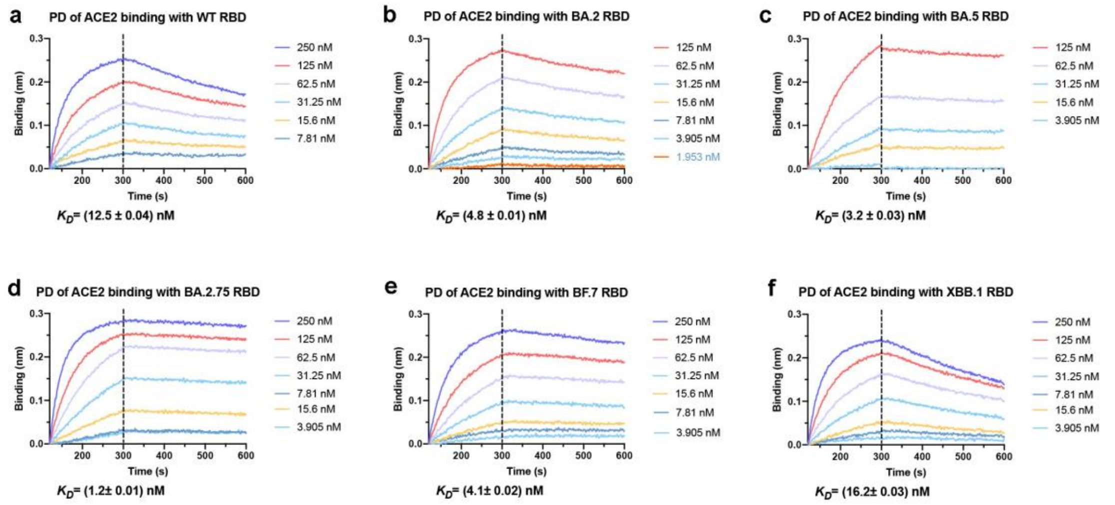

Supplementary Fig. S1 The different affinities of multiple Omicron sub-lineages with the host receptor ACE2.

a-f, Binding affinities of the S-RBD from different Omicron subvariants with the peptidase domain of ACE2 (ACE2-PD). The association and dissociation of S-RBD, applied at different concentrations, with ACE2 PD-coated Streptavidin biosensors was measured using Bio-Layer Interferometry (BLI). K_D was analyzed with Octet Data Analysis HT 12.0 software.

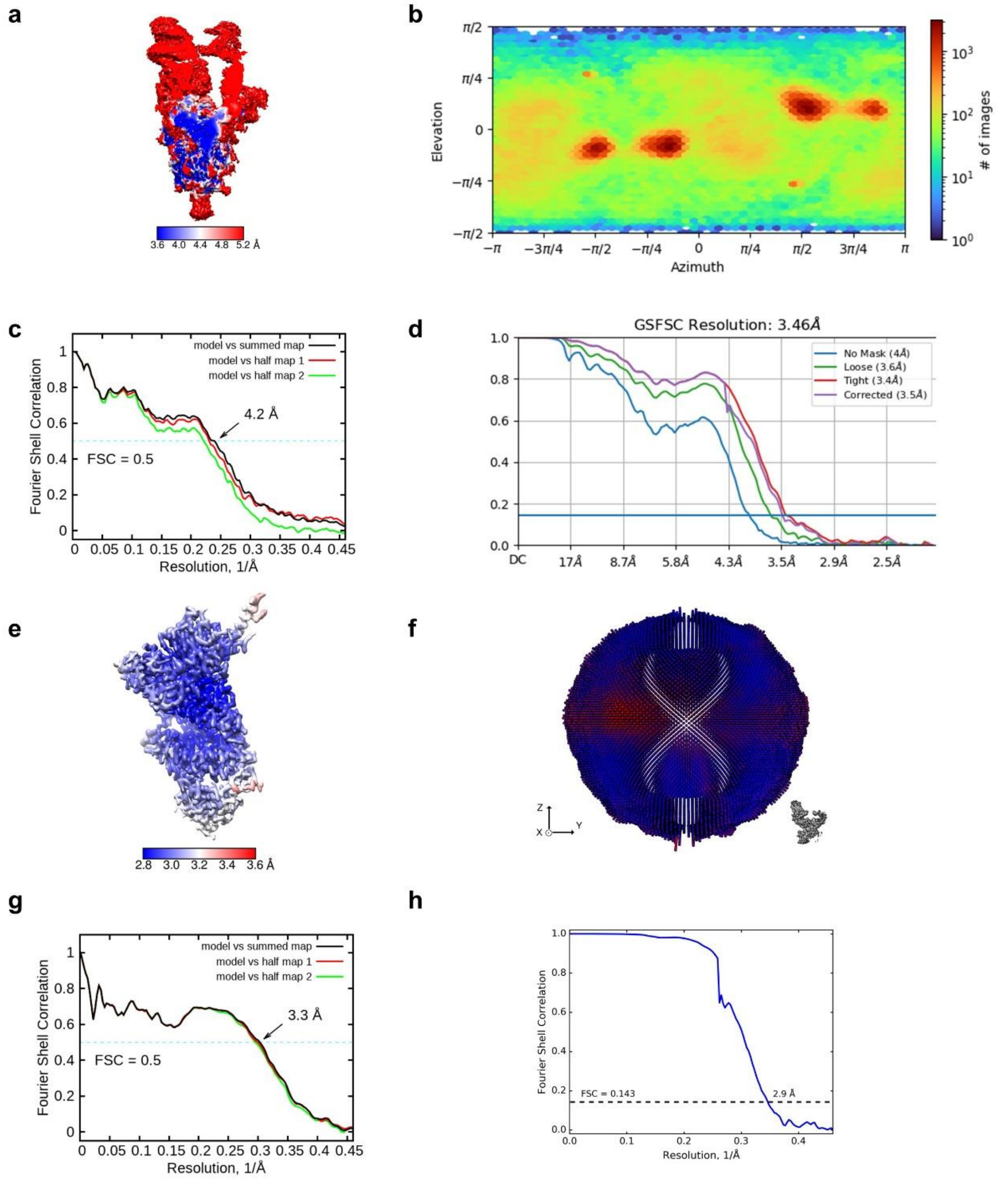

Supplementary Fig. S2 Cryo-EM analysis of S-ECD from BA.2.75-SA.

a, Local resolution map for the 3D reconstruction of the overall structure. **b,** Euler angle distribution in the final 3D reconstruction of overall map. **c,** FSC curve of the refined model of BA.2.75-SA versus the overall structure that it is refined against (black); of the model refined against the first half map versus the same map (red); and of the model refined against the first half map versus the second half map (green). The small difference between the red and green curves indicates that the refinement of the atomic coordinates did not suffer from overfitting. **d,** FSC curve of BA.2.75-SA. **e,** Local

resolution map for the 3D reconstruction of the overall structure and the structure of RBD-PD. **f,** Euler angle distribution in the final 3D reconstruction of RBD-PD sub-complex. **g,** FSC curve of the refined model of RBD-PD sub-complex versus the overall structure that it is refined against (black); of the model refined against the first half map versus the same map (red); and of the model refined against the first half map versus the second half map (green). The small difference between the red and green curves indicates that the refinement of the atomic coordinates did not suffer from overfitting. **h,** FSC curve of RBD-PD sub-complex.

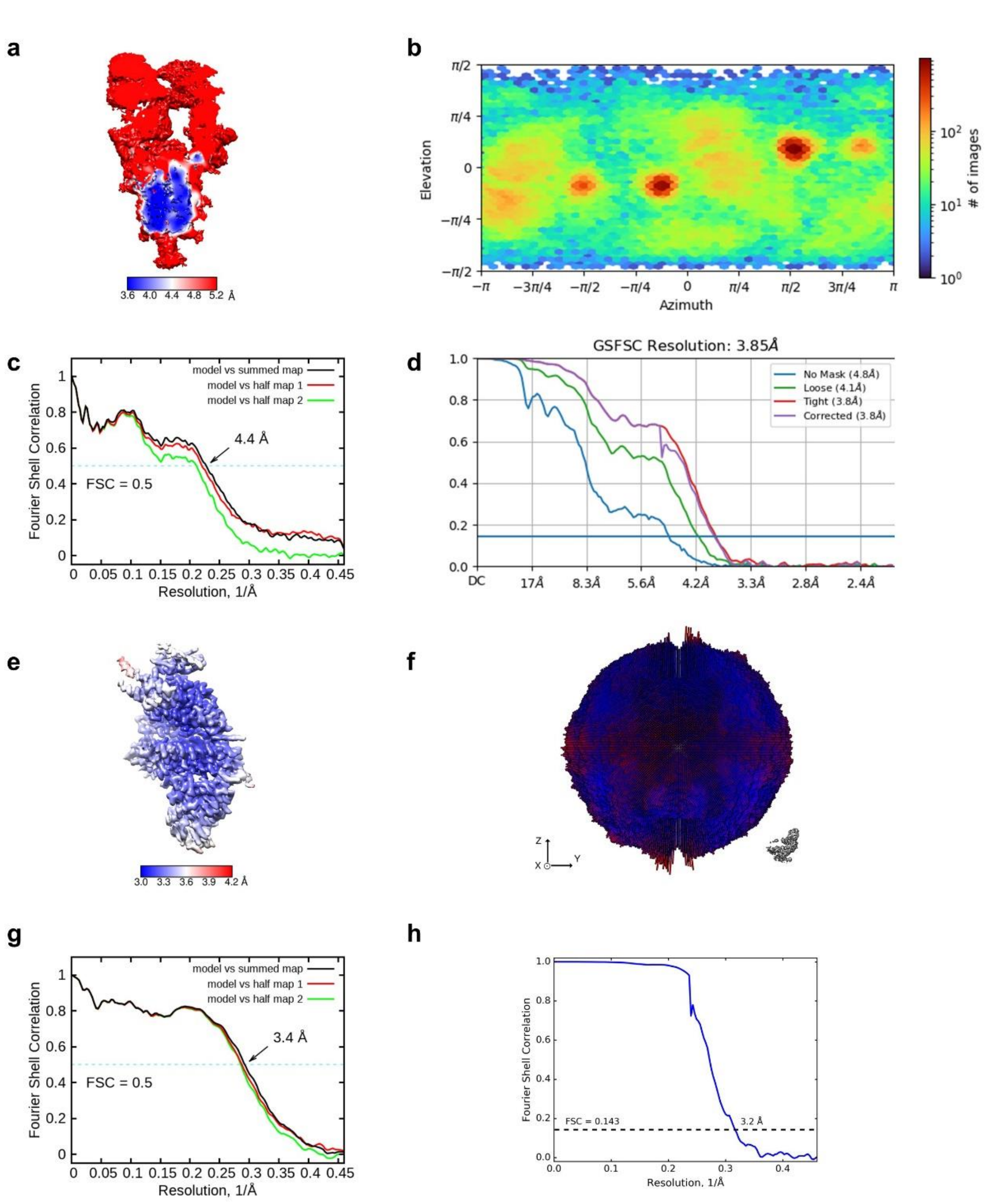

Supplementary Fig. S3 Cryo-EM analysis of S-ECD from BF.7-SA.

a, Local resolution map for the 3D reconstruction of the overall structure. **b,** Euler angle distribution in the final 3D reconstruction of overall map. **c,** FSC curve of the refined model of BF.7-SA versus the overall structure that it is refined against (black); of the model refined against the first half map versus the same map (red); and of the model refined against the first half map versus the second half map (green). The small difference between the red and green curves indicates that the refinement of the atomic

coordinates did not suffer from overfitting. **d,** FSC curve of BF.7-SA. **e,** Local resolution map for the 3D reconstruction of the overall structure and the structure of RBD-PD. **f,** Euler angle distribution in the final 3D reconstruction of RBD-PD sub-complex. **g,** FSC curve of the refined model of RBD-PD sub-complex versus the overall structure that it is refined against (black); of the model refined against the first half map versus the same map (red); and of the model refined against the first half map versus the second half map (green). The small difference between the red and green curves indicates that the refinement of the atomic coordinates did not suffer from overfitting. **h,** FSC curve of RBD-PD sub-complex.

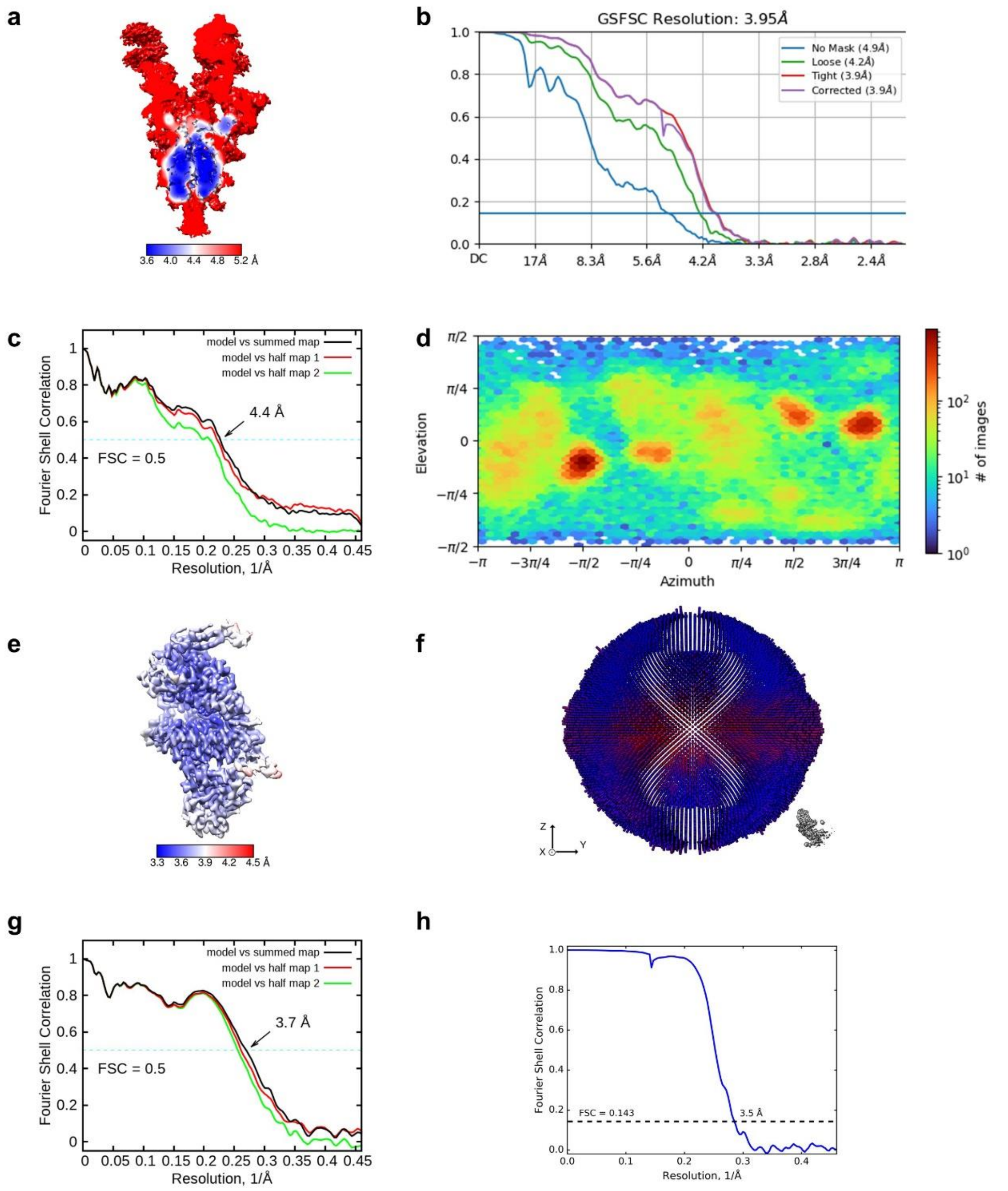

Supplementary Fig. S4 Cryo-EM analysis of S-ECD from XBB.1-SA.

A, Local resolution map for the 3D reconstruction of the overall structure. **B,** Euler angle distribution in the final 3D reconstruction of overall map. **C,** FSC curve of the refined model of XBB.1-SA versus the overall structure that it is refined against (black); of the model refined against the first half map versus the same map (red); and of the model refined against the first half map versus the second half map (green). The small difference between the red and green curves indicates that the refinement of the atomic coordinates did not suffer from overfitting. **D,** FSC curve of XBB.1-SA. **E,** Local

resolution map for the 3D reconstruction of the overall structure and the structure of RBD-PD. **F,** Euler angle distribution in the final 3D reconstruction of RBD-PD sub-complex. **G,** FSC curve of the refined model of RBD-PD sub-complex versus the overall structure that it is refined against (black); of the model refined against the first half map versus the same map (red); and of the model refined against the first half map versus the second half map (green). The small difference between the red and green curves indicates that the refinement of the atomic coordinates did not suffer from overfitting. **H,** FSC curve of RBD-PD sub-complex.

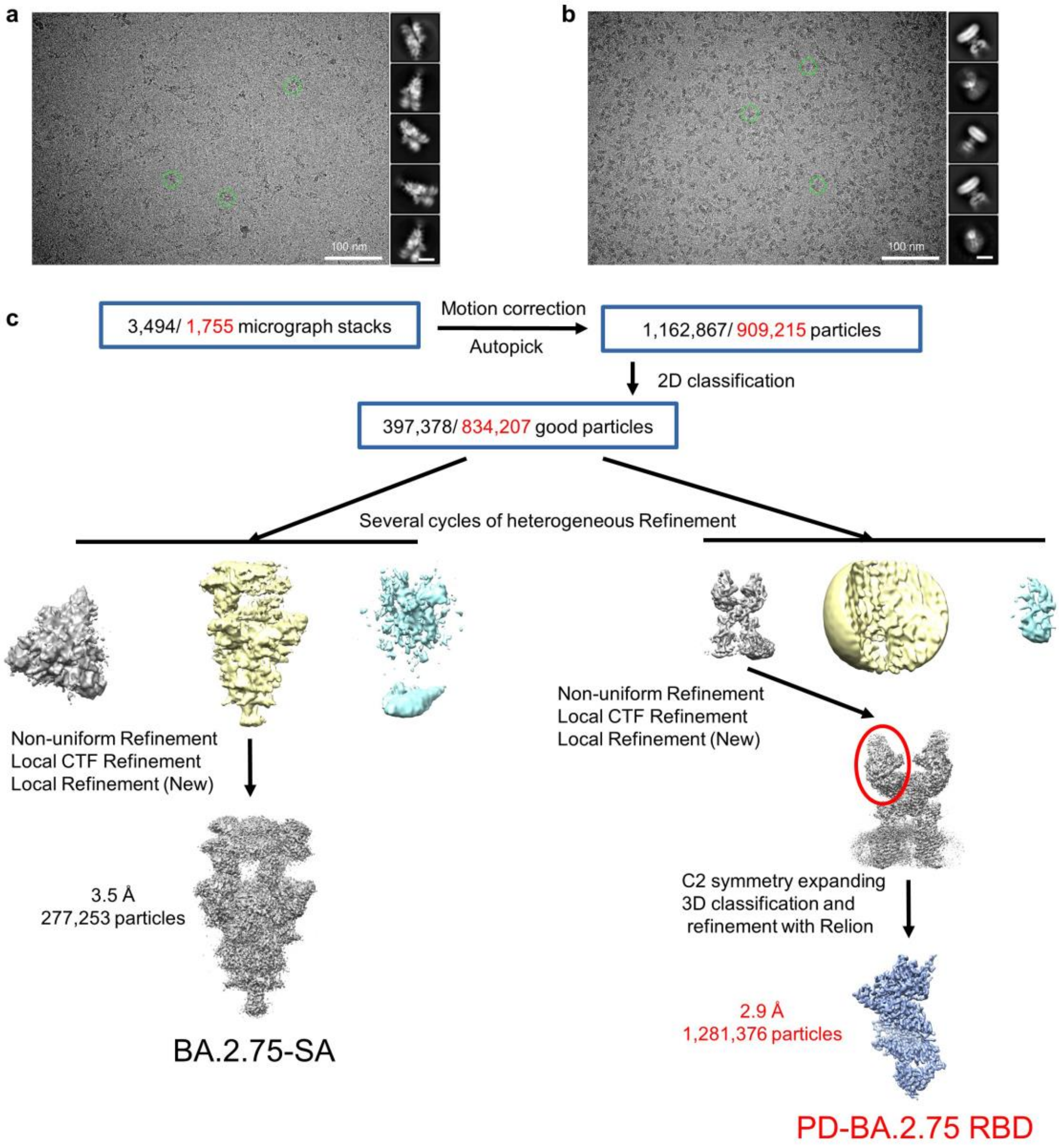

Supplementary Fig. S5 Flowchart of BA.2.75 for cryo-EM data processing.

Please refer to the 'Data Processing' in Methods section for details.

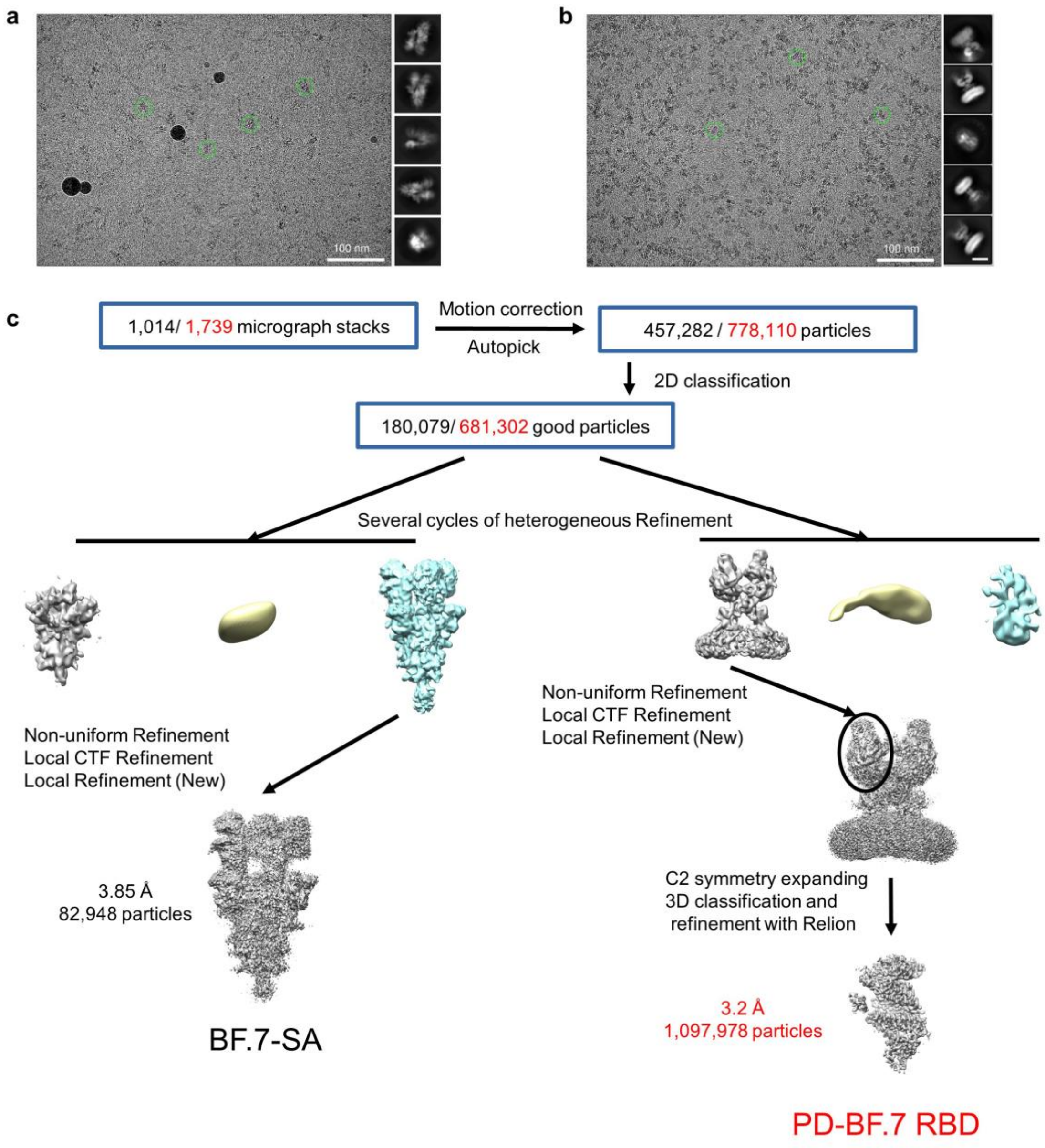

Supplementary Fig. S6 Flowchart of BF.7 for cryo-EM data processing.

Please refer to the ‘Data Processing’ in Methods section for details.

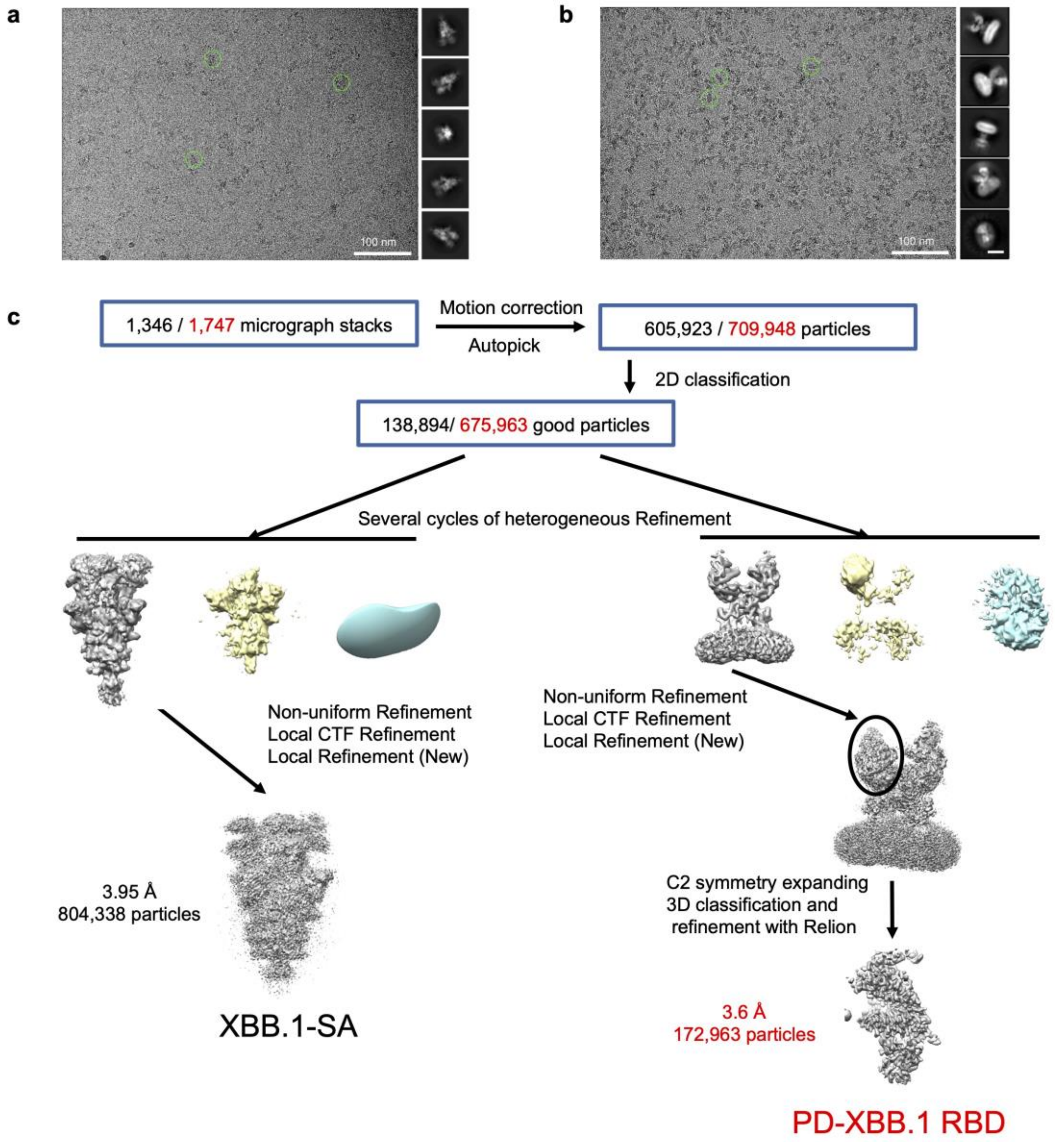

Supplementary Fig. S7 Flowchart of XBB.1 for cryo-EM data processing.

Please refer to the 'Data Processing' in Methods section for details.

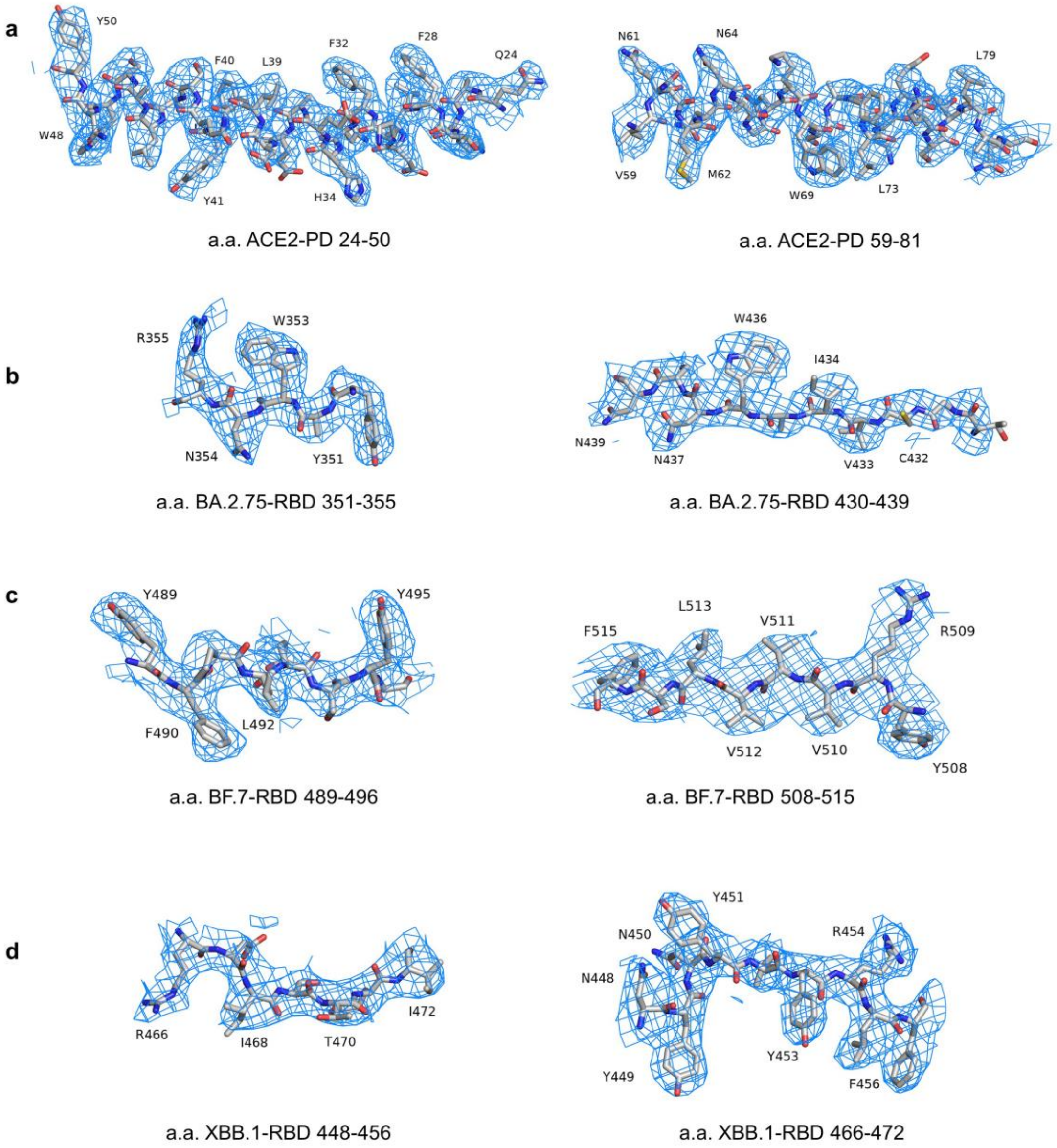

Supplementary Fig. S8 Representative cryo-EM density maps.

a, Cryo-EM density map of ACE2-PD is shown at threshold of 7 σ. **b-d,** Cryo-EM density map of BA.2.75-RBD, BF.7-RBD and XBB.1-RBD are shown at threshold of 7 σ.

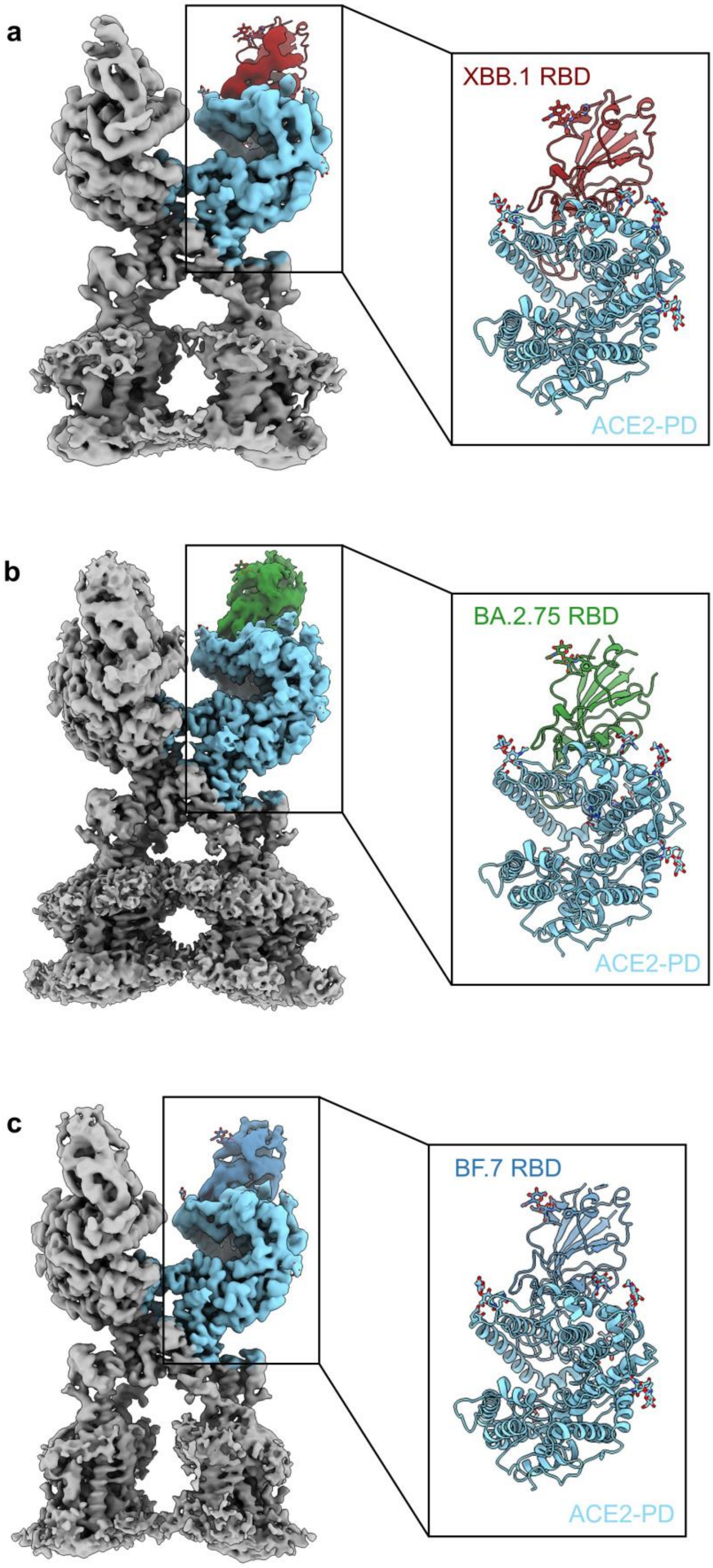

Supplementary Fig. S9 Cryo-EM analysis of SIT1-ACE2 in complex with RBD from spike of Omicron subvariants.

Shown here are the cryo-EM maps of the SIT1-ACE2 in complex with BA.2.75-RBD **(a)**, BF.7-RBD **(b)**, XBB.1-RBD **(c)**, respectively. Insets shows the cartoon presentation of domain-colored cryo-EM structures of S-RBD from Omicron BA.2.75, BF.7, and XBB.1, respectively in complex with the PD of ACE2.

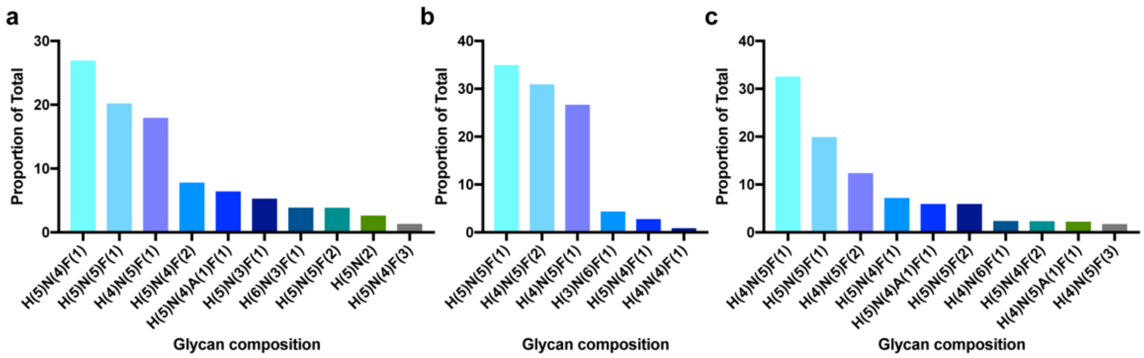

Supplementary Fig. S10 N-linked glycosylation profiles of SARS-CoV-2 S proteins. **a,** Top ten glycan compositions that were identified on trimeric S protein of SARS-CoV-2 WT. **b,** Top ten glycan compositions that were identified on trimeric S protein of SARS-CoV-2 BA.5. **c,** Top ten glycan composition that were identified on trimeric S protein of SARS-CoV-2 XBB.1. H represents hexose; N represents N-acetylglucosamine; F represents fucose; A represents sialic acid.

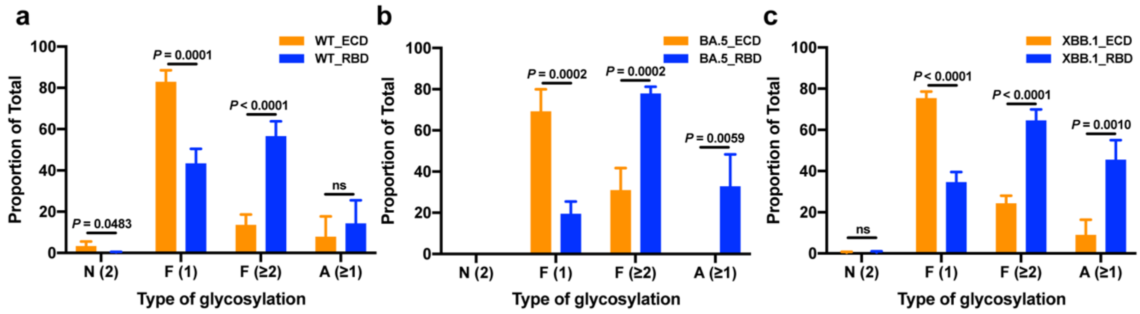

Supplementary Fig. S11 Comparison of N-linked glycosylation profiles between trimeric S protein and monomeric RBD.

a, Comparison of N-linked glycosylation profiles between trimeric S protein and monomeric RBD of SARS-CoV-2 WT. **b,** Comparison of N-linked glycosylation profiles between trimeric S protein and monomeric RBD of SARS-CoV-2 BA.5. **c,** Comparison of N-linked glycosylation profiles between trimeric S protein and monomeric RBD of SARS-CoV-2 XBB.1. N-linked glycans are divided into four categories, including high mannose glycosylation (N2), mono-fucosylation (F1), multi-fucosylation (with ≥ 2 fucose residues, F≥2), and sialylation (A≥1). H represents hexose; N represents N-acetylglucosamine; F represents fucose; A represents sialic acid.

Supplementary Table S1 Cryo-EM data collection and refinement statistics.

**Data collection**		
EM equipment	Titan Krios (Thermo Fisher Scientific)	
Voltage (kV)	300	
Detector	Gatan K3 Summit	
Energy filter	Gatan GIF Quantum, 20 eV slit	
Pixel size (Å)	1.087	1.087
Electron dose (e-/Å2)	50	
Defocus range (μm)	-1.4 ~ -1.8	
Sample	BA.2.75-SA	BA.2.75-RBD-ACE2-SIT1
Number of collected micrographs	3,494	1,755
**3D Reconstruction**		
Software	cryoSPARC	cryoSPARC/Relion
Sample	Overall	BA.2.75-RBD-PD
Number of used particles	277,253	1,281,376
Resolution (Å)	3.5	2.9
Symmetry		
Map sharpening B-factor (Å2)	-90	-90
**Refinement**		
Software	Phenix	
Model composition		
Protein residues	4,786	766
Side chains assigned	4,786	766
Sugar	104	13
R.m.s deviations		
Bonds length (Å)	0.006	0.008
Bonds Angle (˚)	0.982	0.831
Validation		
Clashscore	19.030	4.000
Rotamer outliers (%)	9.830	3.870
Ramachandran plot statistics (%)		
Preferred	91.43	94.95
Allowed	8.14	4.93
Outlier	0.43	0.11

Continued Table

**Data collection**				
EM equipment	Titan Krios (Thermo Fisher Scientific)			
Voltage (kV)	300			
Detector	Gatan K3 Summit			
Energy filter	Gatan GIF Quantum, 20 eV slit			
Pixel size (Å)	1.087	1.087	1.087	1.087
Electron dose (e-/Å2)	50			
Defocus range (μm)	-1.4 ~ -1.8			
Sample	BF.7-SA	BF.7-RBD-ACE2-SIT1	XBB.1-SA	XBB.1-RBD-ACE2-SIT1
Number of collected micrographs	1,014	1,739	1,346	1,747
**3D Reconstruction**				
Software	cryoSPARC	cryoSPARC/Relion	cryoSPARC	cryoSPARC/Relion
Sample	Overall	BF.7-RBD-PD	Overall	XBB.1-RBD-PD
Number of used particles	82,948	1,097,978	804,338	172,963
Resolution (Å)	3.9	3.2	4.0	3.6
Symmetry				
Map sharpening B-factor (Å2)	-90	-90	-90	-90
**Refinement**				
Software	Phenix			
Model composition				
Protein residues	4,786	782	4,786	782
Side chains assigned	4,786	782	4,786	782
Sugar	104	13	104	13
R.m.s deviations				
Bonds length (Å)	0.006	0.007	0.009	0.006
Bonds Angle (°)	1.015	0.819	1.158	0.796
Validation				
Clashscore	14.360	5.030	16.33	8.35
Rotamer outliers (%)	9.03	5.020	9.05	7.43
Ramachandran plot statistics (%)				
Preferred	90.58	93.13	89.42	94.44
Allowed	8.99	6.87	10.19	5.56
Outlier	0.43	0.00	0.39	0.00

Supplementary References:

1 Lei, J. & Frank, J. Automated acquisition of cryo-electron micrographs for single particle reconstruction on an FEI Tecnai electron microscope. *Journal of structural biology* **150**, 69-80, doi:10.1016/j.jsb.2005.01.002 (2005).

2 Zheng, S. Q. *et al.* MotionCor2: anisotropic correction of beam-induced motion for improved cryo-electron microscopy. *Nat Methods* **14**, 331-332, doi:10.1038/nmeth.4193 (2017).

3 Grant, T. & Grigorieff, N. Measuring the optimal exposure for single particle cryo-EM using a 2.6 A reconstruction of rotavirus VP6. *eLife* **4**, e06980, doi:10.7554/eLife.06980 (2015).

4 Zhang, K. Gctf: Real-time CTF determination and correction. *J Struct Biol* **193**, 1-12, doi:10.1016/j.jsb.2015.11.003 (2016).

5 Yaning Li, Q. F., Bing Zhou, Yaping Shen, Yuanyuan Zhang, Lin Cheng, Furong Qi, Shuo Song, Yingying Guo, Renhong Yan, Bin Ju, Zheng Zhang. Structural and functional analysis of an inter-Spike bivalent neutralizing antibody against SARS-CoV-2 variants. *iScience* (2022).

6 Zivanov, J. *et al.* New tools for automated high-resolution cryo-EM structure determination in RELION-3. *Elife* **7**, doi:10.7554/eLife.42166 (2018).

7 Kimanius, D., Forsberg, B. O., Scheres, S. H. & Lindahl, E. Accelerated cryo-EM structure determination with parallelisation using GPUs in RELION-2. *eLife* **5**, doi:10.7554/eLife.18722 (2016).

8 Scheres, S. H. RELION: implementation of a Bayesian approach to cryo-EM structure determination. *Journal of structural biology* **180**, 519-530, doi:10.1016/j.jsb.2012.09.006 (2012).

9 Scheres, S. H. A Bayesian view on cryo-EM structure determination. *Journal of molecular biology* **415**, 406-418, doi:10.1016/j.jmb.2011.11.010 (2012).

10 Punjani, A., Rubinstein, J. L., Fleet, D. J. & Brubaker, M. A. cryoSPARC: algorithms for rapid unsupervised cryo-EM structure determination. *Nature methods* **14**, 290-296, doi:10.1038/nmeth.4169 (2017).

11 Zivanov, J. *et al.* New tools for automated high-resolution cryo-EM structure determination in RELION-3. *eLife* **7**, e42166, doi:10.7554/eLife.42166 (2018).

12 Kimanius, D., Forsberg, B. O., Scheres, S. H. W. & Lindahl, E. Accelerated cryo-EM structure determination with parallelisation using GPUs in RELION-2. *eLife* **5**, e18722, doi:10.7554/eLife.18722 (2016).

13 Scheres, S. H. W. RELION: Implementation of a Bayesian approach to cryo-EM structure determination. *Journal of Structural Biology* **180**, 519-530, doi:https://doi.org/10.1016/j.jsb.2012.09.006 (2012).

14 Scheres, S. H. W. A Bayesian View on Cryo-EM Structure Determination. *Journal of Molecular Biology* **415**, 406-418, doi:https://doi.org/10.1016/j.jmb.2011.11.010 (2012).

15 Rosenthal, P. B. & Henderson, R. Optimal determination of particle orientation, absolute hand, and contrast loss in single-particle electron cryomicroscopy. *Journal of molecular biology* **333**, 721-745 (2003).

16 Chen, S. *et al.* High-resolution noise substitution to measure overfitting and validate resolution in 3D structure determination by single particle electron cryomicroscopy. *Ultramicroscopy* **135**, 24-35, doi:10.1016/j.ultramic.2013.06.004 (2013).

17 Trabuco, L. G., Villa, E., Mitra, K., Frank, J. & Schulten, K. Flexible fitting of atomic structures into electron microscopy maps using molecular dynamics. *Structure (London, England : 1993)* **16**, 673-683, doi:10.1016/j.str.2008.03.005 (2008).

18 Adams, P. D. *et al.* PHENIX: a comprehensive Python-based system for macromolecular structure solution. *Acta crystallographica. Section D, Biological crystallography* **66**, 213-221, doi:10.1107/s0907444909052925 (2010).
